# The association of high Vancomycin trough concentration with acute kidney injury during combination therapy of Piperacillin/Tazobactam and Vancomycin

**DOI:** 10.1016/j.plabm.2022.e00266

**Published:** 2022-01-19

**Authors:** Syuichiro Saito, Takeshi Sugimoto, Kei Takenaka, Hideaki Goto, Akira Kumahara, Rikiya Watanabe, Daisuke Sugiyama, Yoshiro Yasutomo, Kiyonobu Takatsuki

**Affiliations:** a Department of General Internal Medicine & Geriatric Internal Medicine, Japan; b Department of Hematology and Oncology, Japan; c Department of Respiratory Medicine, Kita-Harima Medical Center, Japan; d Department of Division of Pharmacy, Kita-Harima Medical Center, Japan; e Department of Infection Control Committee, Kita-Harima Medical Center, Japan; f Department of Faculty of Nursing and Medical Care, Keio University, Japan

**Keywords:** Acute kidney injury, Piperacillin/Tazobactam, Vancomycin, Trough concentration

## Abstract

**Background:**

Co-administration of Piperacillin/Tazobactam (PIPC/TAZ) and Vancomycin (VCM) as an antibiotic therapy for severe infectious diseases increases the risk of nephrotoxicity. We retrospectively investigated the utility of monitoring VCM trough concentration in early stage of developing acute kidney injury (AKI) on this combination therapy.

**Methods:**

We enrolled all infectious disease patients who were managed with concurrent PIPC/TAZ and VCM. The record of dosage and the administration interval of each antibiotic and its clinical parameters, as well as the VCM trough concentrations, blood culture for bacteria, and serum creatinine values, were collected. VCM trough concentration was measured during the initial 48–72 h of VCM administration. Nephrotoxicity was evaluated as the degree of AKI.

**Results:**

A total of 47 patients fulfilling the criteria were registered, and AKI developed in 10 patients. There was no statistical difference between the AKI and non-AKI groups with regard to age, height, weight, basal creatinine level, body surface area, body mass index, PIPC/TAZ dose, VCM dose, gender, artificial management, and death within around 30 days. The VCM trough level was increased significantly in the AKI group (mean [standard deviation {SD}]: 25.9 [7.8] μg/mL) compared to that in the non-AKI group (mean [SD]: 15.7 [6.9] μg/mL) (p = 0.003). During the clinical course, renal function returned to normal levels in three out of four AKI stage 2 patients, whereas only partial recovery was achieved in all AKI stage 3 patients.

**Conclusions:**

A high VCM trough concentration may have an influence on the occurrence of AKI during combination therapy of PIPC/TAZ and VCM. Careful monitoring of VCM trough concentration will be required to prevent AKI progression.

## 1 Introduction

Co-administration of dual antibiotic therapy for the treatment of infectious diseases is a common clinical practice. Piperacillin/Tazobactam (PIPC/TAZ) and Vancomycin (VCM) are typically co-administered in the context of severe infectious diseases wherein super-infection including beta-lactamase producing bacteria and methicillin-resistant bacteria such as *methicillin-resistant Staphylococcus aureus* (MRSA) is assumed. PIPC/TAZ has rarely been associated with nephrotoxicity in single use [1], whereas high concentrations of VCM carry a risk of nephrotoxicity. Previous studies have investigated whether the risk of acute kidney injury (AKI) increases with co-administration of both antibiotics, and greater AKI risk was associated with combination therapy of PIPC/TAZ and VCM compared to combination therapy of a cephem antibiotic drug and VCM or VCM monotherapy [[2], [3], [4], [5]]. Observational studies have reported that combination therapy of PIPC/TAZ and VCM carries an AKI incidence of around 18%–49% [6]. In fact, this AKI risk has risen according to a recent meta-analysis [7,8]. However, the major risk factor contributing to this nephrotoxicity has been under debate. A VCM steady-state trough concentration of 15 μg/mL or higher is associated with an increased incidence of nephrotoxicity when used in combination with PIPC/TAZ [3]. Recently, nephrotoxicity was usually evaluated as the degree of acute kidney injury (AKI) defined by the Kidney Disease Improving Global Outcomes (KDIGO) criteria. It is fundamentally important to clarify whether high VCM blood concentration really contributes to AKI under combination therapy. In a single-center setting, we retrospectively investigated the utility of monitoring VCM trough concentration in early stage AKI development in the background of PIPC/TAZ and VCM combination therapy.

## 2 Materials and methods

### 2.1 Patients and samples

This was a retrospective cohort study that was reviewed and approved by the Institutional Review Board of Kita-Harima Medical Center (KHMC) in Hyogo, Japan (approval number, 31–48; approval date, February 7, 2020). Patients were recruited from those admitted to KHMC from October 2013 through May 2020 and fulfilled the following criteria: (1) were at least 20 years old, (2) were not receiving renal replacement therapy, (3) had been diagnosed with any infectious disease that is managed by combination therapy of PIPC/TAZ and VCM (if PIPC/TAZ and VCM were administered separately, the administration interval between these two antibiotics should be less than 24 h), (4) had been administered PIPC/TAZ and VCM for 48 h or longer, (5) had known renal function evaluated by serum creatinine (SCr) before antibiotic therapy, and (6) had a known VCM trough concentration during the initial 48–72 h of VCM administration. Only the data of VCM trough concentration collected immediately before next VCM administration was considered as valid. If VCM trough concentration was measured more than once within the mentioned period, the data with higher concentration value was used for analysis.

Data about the administration of antibiotics (dosage, administration interval, and administration days) were primarily collected from the antibiotics usage monitoring data of the infection control team in KHMC and re-confirmed using the antibiotics usage certification record on an electronic medical record (EMR). Patient demographics and other information such as age, gender, height, weight, body mass index (BMI), body surface area (BSA), type of infection, underlying disease, and presence or absence of support of artificial respiration were recorded through EMR review as well as the laboratory data (VCM trough concentration, blood culture test for bacteria, and SCr value). The SCr was determined by enzymatic method, and the VCM trough concentration was determined by chemiluminescent immunoassay method. Infection control committee in KHMC recommended that the maximum daily dose of PIPC/TAZ and VCM were 18g and 2g, respectively. The daily dose of these antibiotics was actually determined by the attending physicians.

### 2.2 Evaluation of nephrotoxicity

AKI was defined as nephrotoxicity after the concomitant administration of PIPC/TAZ and VCM using the KDIGO SCr stage criteria [9], as follows: Stage 1 (increase in SCr ≥0.3 mg/dL within 48 h or 1.5–1.9 times the baseline), stage 2 (increase in SCr 2.0–2.9 times the baseline), and stage 3 (increase in SCr 3.0 times the baseline or to ≥4.0 mg/dL or initiation of renal replacement therapy). Renal function was evaluated in each patient using only the SCr index part of KDIGO criteria and did not consider the urine output criteria. For this study, the AKI diagnosis was defined by being stage 1 or greater based on KDIGO criteria. Reference interval of SCr was defined as 0.6–1.1 mg/dL in males and 0.4–0.9 mg/dL in females, because of the gender differences in normal SCr value.

### 2.3 Monitoring interval in patients with nephrotoxicity

Day 1 of each patient was set as day that the second antibiotic (VCM) was administered and clinical course was monitored until day 30 or until the patient expired. The information of a patient’s status and clinical course, as well as the SCr value, was extracted from EMR. Recovery of renal function was classified as three levels: full recovery (absence of AKI criteria), partial recovery (fall in AKI stage), and non-recovery (maintenance of AKI stage) [10]. Recovery of renal function was assessed with the best SCr value after the treatment for AKI until day 30 or until the patient expired.

### 2.4 Statistical analysis

All of the statistical analyses were performed using the Japanese version of KaleidaGraph™ 4 (Hulinks©). Comparisons of values regarding each item were calculated using Student's t-test or Fisher’s exact probability test. Values were expressed as “mean SD.” In all analyses, p values < 0.05 were considered to be statistically significant, and all statistical tests were two-sided.

## 3 Results

A total of 59 patients received combination therapy of PIPC/TAZ and VCM during the study period, and 47 patients fulfilled the criteria and were thus registered (Fig. supple 1). Clinical characteristics of patients upon being enrolled are listed in Table 1. Blood culture samples were submitted for 41 patients, with 19 patients (46%) having at least one positive sample, and MRSA was detected in 13 patients. A total of nine patients (19%) expired around 30 days, wherein eight patients died before day 30, and one patient died on day 33. In this cohort, AKI developed in 10 patients (21%) based on the KDIGO criteria (Fig. supple 1). Results of a comparative analysis between the AKI and non-AKI groups expressed as mean (SD), respectively and calculated using the *t*-test are presented in Table 1. Overall, we did not find any statistical difference in these factors between the two groups, even though the AKI group had higher rates of positive blood culture than the non-AKI group (p = 0.12).

**Table 1:** Patient characteristics and statistical analysis between the AKI and non-AKI groups.

	All	AKI	non-AKI	*p* value
Infectious disease (n)	47	10	37	
Pneumonia	23	7	16	
Sepsis	6	0	6	
Febrile neutropenia	6	2	4	
Infection in the perioperative period	5	1	4	
Cellulitis	3	0	3	
Pyogenic spondylitis	2	0	2	
Infection with critical limb ischemia	1	0	1	
Biliary tract infection	1	0	1	
Male/Female (n)	32/15	7/3	25/12	1.0^c^
Age a	73.8 (12.7)	72.1 (14.3)	74.3 (12.4)	0.66 b
Height (cm) a	159.4 (9.4)	159.4 (11.1)	159.3 (9.1	0.99 b
Weight (kg) a	56.7 (15.2)	58.9 (15.1)	56.1 (15.4)	0.61 b
Basel creatinine (mg/dl) a	0.90 (0.43)	0.84 (0.38)	0.91 (0.44)	0.65 b
Body surface area (m^2^) a	1.56 (0.21)	1.56 (0.22)	1.56 (0.20)	0.95 b
Body mass index a	22.2 (6.3)	22.5 (7.9)	22.1 (5.9)	0.89 b
Blood culture test, positive/negative (n)	19/22	6/2	13/20	0.12^c^
Artificial respirator management, yes/no (n)	5/42	2/8	3/34	0.29^c^
Death within 30 days, yes/no (n)	9/38	3/7	6/31	0.38^c^
PIPC/TAZ dose (g/day) a	12.9 (3.2)	12.4 (4.4)	13.0 (2.8)	0.67 b
Vancomycin dose (g/day) a	1.16 (0.50)	1.25 (0.72)	1.14 (0.43)	0.65 b

a Mean (Standard deviation). *P*-value was calculated by.

b *t*-test and.

c Fisher’s exact probability test.

**Fig. 1:**
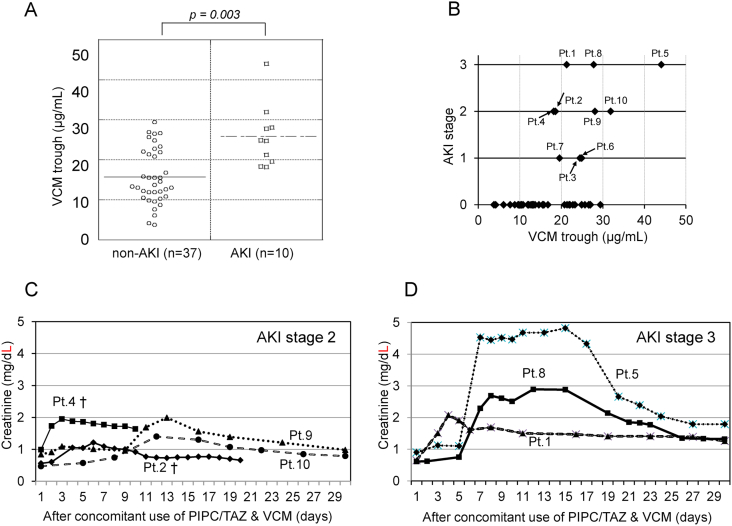
(A) Plot data of the vancomycin (VCM) trough level in the non-acute kidney injury (non-AKI) group and the AKI group. The VCM trough level was significantly increased in the AKI group.(B) Plot data of the VCM trough level according to AKI stage.(C) Transition of the serum creatinine (SCr) value after concomitant use of PIPC/TAZ and VCM in AKI stage 2 patients (n = 4). Patients who died within 30 days marked with a dagger (†).(D) Transition of the SCr value after concomitant use of PIPC/TAZ and VCM in AKI stage 3 patients (n = 3). AKI, evaluated using the serum creatinine value, developed at around day 7 and persisted for one week in patients 5 and 8. Renal function in each of the three cases improved gradually after AKI and reached partial remission around day 30. Abbreviation: AKI, acute kidney injury; PIPC/TAZ, Piperacillin/Tazobactam; VCM, Vancomycin; Pt., patient. (B, C, D) Each patient No. corresponds to the patient No. listed in Table 2.

Next, we looked into 10 patients who developed AKI after antibiotic therapy. Table 2 lists the characteristics of these patients. Basal SCr values at the time of therapy initiation were normal in eight patients and abnormal in two patients (patients 3 and 7). All 10 patients were classified into AKI stages in reference to the peak SCr value as follows: Three patients were stage 1; four patients were stage 2; and three patients were stage 3 (Table 2). VCM was then stopped or reduced in dosage after the development of AKI in these patients. The renal function returned to normal (full recovery) in three patients, while four patients only partially recovered; gender differences were considered in defining normal creatinine values. Two patients died without renal recovery, and another patient (patient 7) had no improvement in the SCr value (non-recovery). Next, the relationship between the VCM trough concentration and nephrotoxicity were investigated through comparison of the AKI and non-AKI groups. The VCM trough level was significantly greater in the AKI group (mean [SD], 25.9 [7.8] μg/mL) compared to that in the non-AKI group (15.7 [6.9] μg/mL) (p = 0.003) (Fig. 1-A). The distribution of VCM trough level and AKI stage was plotted in Fig. 1-B.

**Table 2:** Characteristics of ten patients who had acute kidney injury after concomitant usage of PIPC/TAZ and VCM.

Patient No.	Age/gender	Diagnosis of infection	Basal diseases	PIPC/TAZ dose (g/day)	VCM dose (g/day)	Basal SCr (mg/dl)	Peak SCr (mg/dl)	aOutcome SCr (mg/dl)	⁺Time to onset of AKI (days)	AKI stage (KDIGO criteria)	Renal Outcome
1	66/F	surgical site infection	Post surgery for inguinal hernia	13.5	2.5	0.65	2.08	1.24	1	3	Partial recovery
2	78/M	Aspiration pneumonia	Pneumothorax	13.5	1.5	0.56	1.21	0.66 (day20)	2	2	Full recovery (death on day23)
3	62/M	Aspiration pneumonia	Brain hemorrhage	6.75	0.75	1.11	1.98	1.43	2	1	Partial recovery
4	79/M	Aspiration pneumonia	Post surgery for gastric cancer	4.5	1.5	0.94	1.95	1.64 (day11)	1	2	Non-recovery (death on day11)
5	51/M	Febrile pneutropenia	Chemotherapy for APL	18	2	0.9	4.92	1.79	5	3	Partial recovery
6	85/M	Aspiration pneumonia	Cerebral infarction	13.5	0.5	0.56	1.08	1.08 (day12)	6	1	Non-recovery (death on day12)
7	87/F	Aspiration pneumonia	Chronic heart failure	13.5	0.5	1.75	3.35	3.3	8	1	Non-recovery
8	87/M	Aspiration pneumonia	Traumatic cerebral hemorrhage	13.5	1	0.66	2.89	1.32	5	3	Partial recovery
9	49/M	Febrile neutropenia	Chemotherapy for APL	18	2	0.84	1.99	0.98	9	2	Full recovery
10	77/F	Aspiration pneumonia	Acute pyelonephritis	13.5	2	0.47	1.4	0.79	10	2	Full recovery

Abbreviation: APL, acute promyelocytic leukemia; AKI, acute kidney injury; SCr, serum creatinine.

a Outcome SCr was the value obtained at the first blood test on day 30 or later from concomitant use of PIPC/TAZ and VCM. As the evaluation of renal outcome on the patients who died before day 30 (patient 2, 4, and 6), we determined based on last SCr value. ⁺Time to onset of AKI was the time between initial VCM administration and onset of AKI.

AKI patients that recovered their renal function throughout their clinical course were compared based on the 30 days mortality endpoint and renal outcome. SCr values were analyzed starting from the initiation of combination therapy to approximately 30 days after the treatment for the four patients classified as AKI stage 2 and for the three patients classified as AKI stage 3. SCr values returned to normal (full recovery) in three of the four patients in AKI stage 2. The exception was patient 4 who succumbed to respiratory failure on day 11 due to aspiration pneumonia (Fig. 1-C and Table 2). By contrast, the SCr peak value declined in the three patients in AKI stage 3. SCr values improved two weeks after starting concomitant antibiotic therapy in two patients (patients 5 and 8). Renal function in these three patients improved partially without returning to normal on day 30 (partial recovery) (Fig. 1-D and Table 2). Regarding the time to onset of AKI, AKI was developed for 5 days or more after VCM administration in two of AKI stage 2 cases (patient 9 and 10) and in two of AKI stage 3 cases (patient 5 and 8).

## 4 Discussion

The risk factors for the development of nephrotoxicity during combination therapy of PIPC/TAZ and VCM are still under discussion, and the reason behind their associated higher AKI development rates is not clearly understood. One possible reason is that these antibiotics have an additive effect that causes the occurrence of acute interstitial nephritis or direct cellular necrosis [11]. Another explanation is that PIPC/TAZ decreases the clearance of VCM, resulting in an accumulation of VCM in the body [3,4,11]. Our results indicated that VCM trough concentration was significantly higher in the AKI group compared to that in the non-AKI group, although the two groups did not differ in terms of antibiotic dose and the fundamental indices of basal SCr, BSA, and BMI. Therefore, we speculate that higher VCM trough concentrations may be associated with nephrotoxicity in the context of VCM and PIPC/TAZ combination therapy. High serum concentrations of VCM as monotherapy have previously been associated with AKI. However, combination therapy with PIPC/TAZ seems to play a role in suppressing the renal excretion of VCM by an unknown mechanism, resulting in higher levels of VCM concentration. It is still unclear whether the high VCM trough concentration is the cause or result of this AKI. However, in the presenting data, AKI was occurred at 5 or more days after VCM administration in nearly half of AKI stage 2 and 3 parents. This result implies that high VCM concentration is contributory to AKI development.

Regarding the management of VCM concentration, periodic monitoring of VCM trough levels is recommended to prevent the onset of nephrotoxicity. In practice, the target trough level is set to 15–20 μg/mL for optimal therapeutic outcomes [12]. However, a VCM trough concentration of 15 μg/mL or higher has been reported as risk for AKI [3,13,14]. Because of these contradictory findings, it remains unknown whether a lower (10–15 μg/mL) or a higher (15–20 μg/mL) VCM trough level is truly optimal [15]. A general consensus has been reached, however, that the risk of nephrotoxicity increases with a VCM trough level of 20 μg/mL or higher [[16], [17], [18]]. In our study, only a few patients with a VCM trough level below 20 μg/mL developed AKI [Fig. 1-A]. Our findings are consistent with a report from Burgess et al., wherein AKI incidence was associated with VCM steady-state trough concentration in the context of combination therapy with PIPC/TAZ [3]. A few studies have reported that there is no association between VCM trough concentration and incidence of AKI during combination therapy with PIPC/TAZ [19]; thus, further study on this is warranted. Our results suggest that VCM trough concentration should be kept below 20 μg/mL to prevent the occurrence of AKI in the context of combination therapy with PIPC/TAZ. In our study, VCM trough concentrations over 20 μg/mL were observed in 19 (40%) out of 47 patients. We recognize that more careful monitoring for VCM concentration is required, and it should be decreased promptly if it exceeds 20 μg/mL.

In this study, most patients who developed stage 1 or 2 AKI achieved full recovery of renal function with adequate VCM trough management, assuming they had normal baseline SCr values. By contrast, two patients (patients 3 and 7) with abnormal basal SCr values who developed stage 1 AKI still had renal dysfunction even after treatment. Thus, PIPC/TAZ and VCM combination therapy should be avoided in patients with abnormal basal SCr values. Among the three patients who developed AKI stage 3, renal function recovered only partially on day 30, indicating the difficulty of complete repair after severe renal damage these antibiotics. We observed that renal dysfunction persisted for one week and improved gradually afterwards in two cases (patient 5 and 8) upon SCr monitoring of stage 3 AKI (Fig. 1-D). It is interesting how a significant level of renal recovery can still be expected even in cases of stage 3 AKI. To obtain a good renal prognosis, early interventions upon the occurrence of AKI, such as altering antibiotics while managing the patient’s general condition, are necessary. Since there are limited reports describing the severity of AKI and its clinical course, our results can be useful as a reference for AKI treatment using the same antibiotics.

This study has several limitations. First, this is a retrospective study conducted with a limited sample cohort at a single center. Second, the dosage and duration of administration of PIPC/TAZ and VCM was scheduled by each attending physician, which might cause variations between patients. Third, blood sampling to measure VCM trough levels was done depending on each attending physician’s schedule, resulting in variations regarding the count and time interval between VCM trough checking times in individual patients. Lastly, there was no standardized plan for reducing antibiotics after the occurrence of nephrotoxicity. However, there are limited existing data about the transition of renal function during the clinical course of AKI in the context of PIPC/TAZ and VCM combination therapy; thus, our data will prove useful. More data on the management of VCM administration before and after the onset of AKI are required.

## 5 Conclusions

A high VCM trough concentration may have an influence on the occurrence of AKI during combination therapy of PIPC/TAZ and VCM. Also, it is speculated that the rise of VCM trough concentration is accelerated under AKI status. Controlling an excess VCM trough concentration seems to be useful for preventing the development of AKI. If we come across the early stage of developing AKI, prompt check of VCM trough concentration will be required in this combination therapy.

## Declaration

The authors state that they have no Conflict of Interest.

## Authors’ contribution

SS and TS designed and performed the analysis of data. KeT and HG supported the analysis of occurring AKI cases. AK proposed the optimal dosage and interval of VCM administration. RW reviewed the manuscript, and DS performed the statistical analysis. YY and KiT supervised this study.

## Funding

The authors did not receive any funding.

## Declaration of competing interest

☑ All authors have participated in (a) conception and design, or analysis and interpretation of the data; (b) drafting the article or revising it critically for important intellectual content; and (c) approval of the final version.

☑ This manuscript has not been submitted to, nor is under review at, another journal or other publishing venue.

☑ The authors have no affiliation with any organization with a direct or indirect financial interest in the subject matter discussed in the manuscript.

☑ The following authors have affiliations with organizations with direct or indirect financial interest in the subject matter discussed in the manuscript.
