# Supplementary material for: The association of high Vancomycin trough concentration with acute kidney injury during combination therapy of Piperacillin/Tazobactam and Vancomycin
Source: Pract Lab Med. 2022 Jan 19;29:e00266. doi: 10.1016/j.plabm.2022.e00266 (PMC8789664; doi:10.1016/j.plabm.2022.e00266)
Supplement: Multimedia component 1 [file mmc1.pptx]

## Slide 1
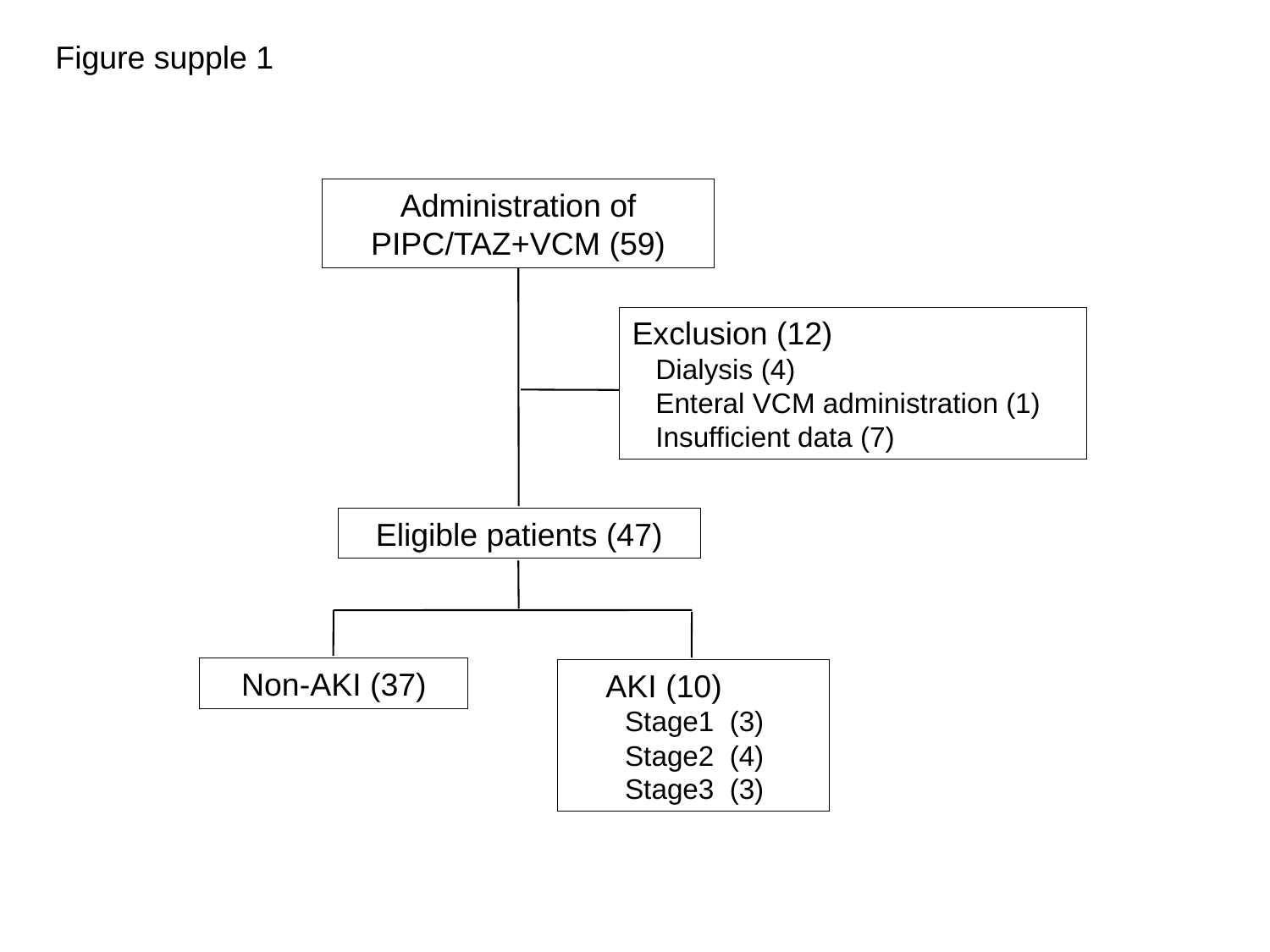

Figure supple 1
Administration of PIPC/TAZ+VCM (59)
Exclusion (12)
 Dialysis (4)
 Enteral VCM administration (1)
 Insufficient data (7)
Eligible patients (47)
Non-AKI (37)
 AKI (10)
 Stage1 (3)
 Stage2 (4)
 Stage3 (3)
